# Water-Driven Sol–Gel Transition in Native Cellulose/1-Ethyl-3-methylimidazolium Acetate Solutions

**DOI:** 10.1021/acsmacrolett.3c00710

**Published:** 2024-01-29

**Authors:** Roshan Akdar Mohamed Yunus, Marcus Koch, Philippe Dieudonné-George, Domenico Truzzolillo, Ralph H. Colby, Daniele Parisi

**Affiliations:** † Engineering and Technology Institute Groningen (ENTEG), University of Groningen, Nijenborgh 4, 9747 AG Groningen, The Netherlands; ‡ INM − Leibniz Institute for New Materials, Campus D2 2, 66123 Saarbrücken, Germany; § Laboratoire Charles Coulomb (L2C), UMR 5221 CNRS Université de Montpellier, Montpellier 34095, France; ∥ Department of Materials Science and Engineering, Penn State University, University Park, Pennsylvania 16802, United States

## Abstract

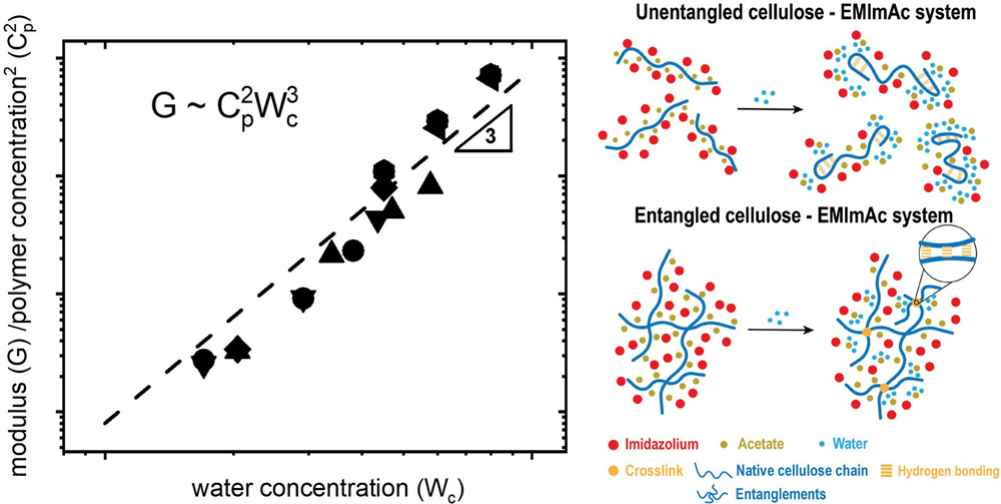

The addition of water to native cellulose/1-ethyl-3-methylimidazolium acetate solutions catalyzes the formation of gels, where polymer chain–chain intermolecular associations act as cross-links. However, the relationship between water content (*W*_c_), polymer concentration (*C*_p_), and gel strength is still missing. This study provides the fundamentals to design water-induced gels. First, the sol–gel transition occurs exclusively in entangled solutions, while in unentangled ones, intramolecular associations hamper interchain cross-linking, preventing the gel formation. In entangled systems, the addition of water has a dual impact: at low water concentrations, the gel modulus is water-independent and controlled by entanglements. As water increases, more cross-links per chain than entanglements emerge, causing the modulus of the gel to scale as *G*_p_ ∼ *C*_p_^2^*W*_c_^3.0±0.2^. Immersing the solutions in water yields hydrogels with noncrystalline, aggregate-rich structures. Such water–ionic liquid exchange is examined via Raman, FTIR, and WAXS. Our findings provide avenues for designing biogels with desired rheological properties.

Native cellulose is the most plentiful biopolymer on Earth,^1^ produced by nature in hundreds of billions of tons annually.^2,3^ However, native cellulose cannot be melt-processed and, due to inter- and intramolecular hydrogen bonding between cellulose chains, is insoluble in water,^4^ limiting its exploitation in industry and biomedicine.

Native cellulose dissolves at the molecular level in some ionic liquids (ILs),^5,6^ e.g., 1-ethyl-3-methylimidazolium acetate (EMImAc).^7^ The solvation of native cellulose in IL is significantly dominated by hydrogen bonds between the anions and the polar domains of the native cellulose chains.^8^ Owing to the similar dimensions between the imidazolium rings and the distance of consecutive nonpolar groups of the cellulose chains, the cations intercalate within these sites, favoring van der Waals cation–cellulose interactions.^9^ The positive charge present at each end of the imidazolium rings of the cations promotes the formation of cohesive anion–cation sequences at the surface of cellulose chains, therefore preventing intercellulose hydrogen bond interactions and favoring molecular dissolution of native cellulose. This discovery enabled the processing of cellulose in solution,^10−13^ leading to numerous applications in a variety of fields, including catalysis,^14^ electrochemistry,^15^ polymer chemistry,^16^ fiberspinning,^13^ and electrospinning.^17^ As a matter of fact, various solvents have been found able to dissolve cellulose.^18^ However, the debate of whether they derivatize native cellulose is far from being clarified, in addition to other major issues: thermal and chemical instabilities, oxidative side reactions, prohibitive costs, need for low temperature (−5 °C) or acidic conditions for the dissolution, toxicity, and difficult preparation methods.^18−20^ Conversely, the nonderivatizing EMImAc has been found thermally and chemically stable and is even considered suitable for designing gels for biomedical applications.^21−24^

Over the years, several authors have reported about cellulose/EMImAc/water interactions^25−27^ and, in particular, the sol–gel transition of cellulose/IL solutions driven by water absorption.^28−34^ This phenomenon has also been exploited to regenerate cellulose.^18,30^ A proposed mechanism for the sol–gel transition is based on the water–ionic liquid and cellulose–ionic liquid hydrogen bonding competition.^32^ As water is added into the solution, strong binding between water molecules and the acetate anions occurs.^35^ The ion–pair and polymer chain–ionic liquid interactions are disrupted by the presence of water molecules, freeing the hydroxyl groups of the polymer chains, which in turn drive intercellulose hydrogen bonds. Such intermolecular associations act as effective cross-links in the system, favoring the formation of a three-dimensional network. While the effect of water on cellulose in ionic liquid solutions has been already reported,^28−34^ the correlation between polymer content, water concentration, and gel strength is marginally explored. This work delves deeper into the role of water on the rheostructural properties of gels formed in ternary native cellulose/EMImAc/water systems. Water will be used as an elegant albeit unusual cross-linking agent for native cellulose gels. This novel rheology toolbox will boost the use of water to better control native cellulose and its processing, which is pivotal for the current large demand for precisely controlled and easily controllable soft (bio)materials. A combination of linear shear rheology, Raman and Fourier-transform infrared (FTIR) spectroscopy, cryogenic transmission electron microscopy (cryo-TEM), and wide-angle X-ray scattering (WAXS), was adopted to shed light on the sol–gel transition of native cellulose/ionic liquid solutions.

Figure 1 shows the linear viscoelastic spectra of native cellulose in EMImAc solutions at different polymer concentrations of *C*_p_ and water contents *W*_c_. In dry conditions, such systems behave as linear flexible polymer solutions,^36,37^ with an entanglement plateau for polymer contents above the entanglement concentration *C*_e_, and a terminal flow (*G*′ ∼ ω^2^, *G*″ ≫ *G*′) at low frequencies. The latter reflects the molecular dissolution of the native cellulose in an ionic liquid, as observed in other works.^31,36−38^ As water is introduced into the system, the rheological response of the solutions strongly depends on the initial polymer concentration. The overlap C* and entanglement concentrations of the native cellulose (molar mass *M* = 625 kg/mol) in EMImAc are 0.15 and 0.85 wt %, respectively (see Figure S1 in the Supporting Information (SI)), in agreement with previously reported values.^39^ For *C*_p_ ≤ C*, an increase in water concentration translates into a decrease of the moduli (and the zero-shear viscosity; Figure 1A). In the unentangled regime, polymer chains reduce their coil size, likely due to the worsening of the solvency conditions with the addition of water and possible intramolecular associations. According to the Zimm model,^40^ the ratio η_sp_/*C*_p_ is proportional to *R*^3^, where η_sp_ and *R* represent the specific viscosity and the chain size, respectively. The inset in Figure 1A shows a decrease in η_sp_/*C*_p_ with increasing water content, reflecting a chain size reduction (see discussion in SI). As soon as an entangled polymer network is formed (*C*_p_ ∼ *C*_e_), a water content as low as 6 wt % suffices to dynamically arrest the solution (Figure 1B). A viscoelastic fluid that does not show flow behavior within 100 s (0.01 rad/s) is considered to be a gel.^41−45^ Further addition of water promotes an increase in the gel strength (Figure 1B). For larger polymer contents (Figure 1C), the amount of water needed to form a gel decreases. It is also important to note that, with 40 wt % of water, the modulus of the gel increases by more than 1 order of magnitude.

**Figure 1:**
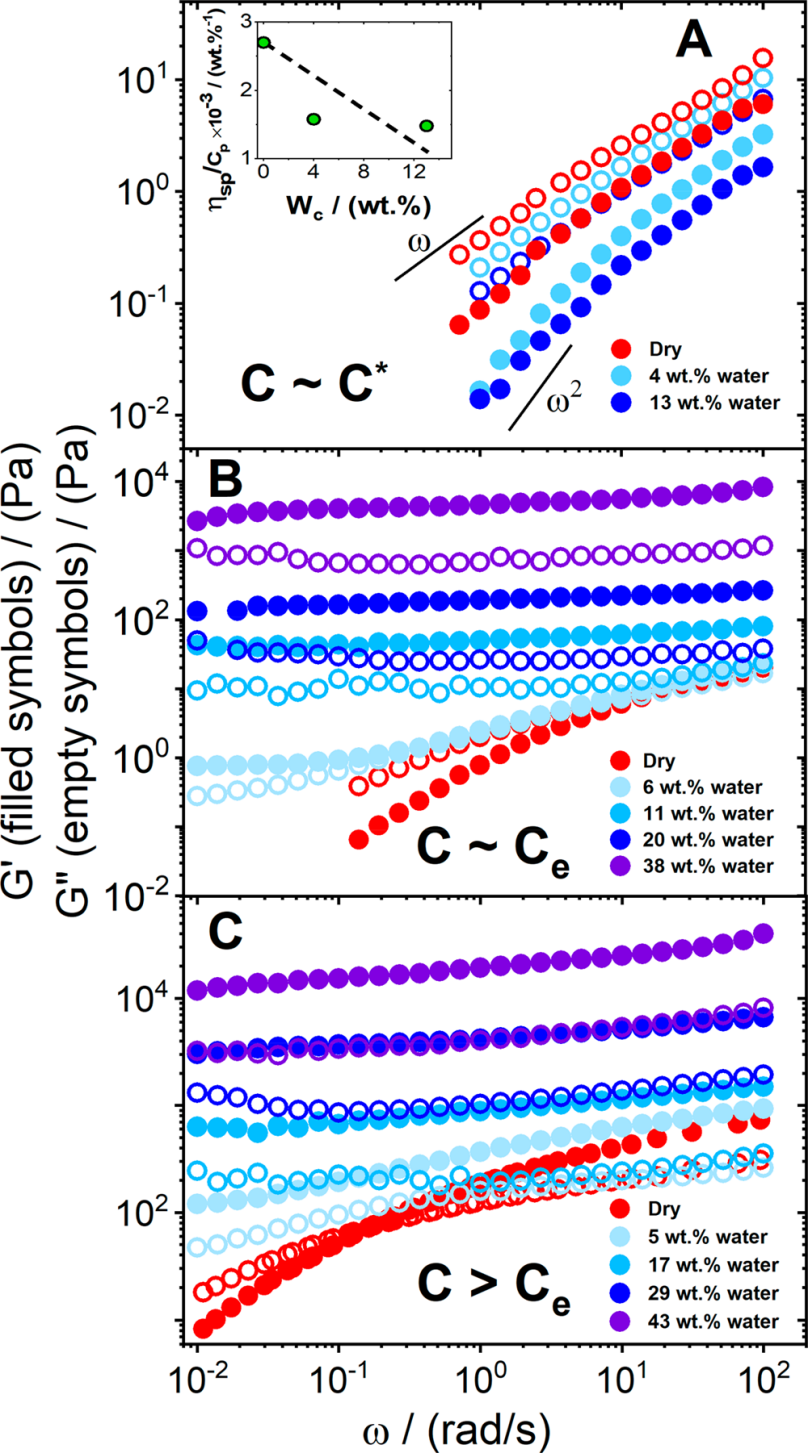
Storage *G*′ (closed symbols) and loss *G*″ (open symbols) moduli as a function of oscillation frequency ω for native cellulose/EMImAc solutions at various polymer and water mass fractions. The polymer concentration *C*_p_ varies from the overlap concentration C* (0.1 wt %, panel A) and the entanglement concentration *C*_e_ (0.8 wt %, panel B, to 2 wt %, panel C) within the entanglement regime. The water content is reported in the legend of each panel. The solid lines in panel A represent the expected slopes for the dynamic moduli in the terminal regime (Newtonian behavior). The inset in panel A reports the specific viscosity divided by the polymer concentration (η_sp_/*C*_p_) as a function of the water concentration (*W*_c_). The dashed line is a guide for the eye. A decreasing trend with increasing *W*_c_ values can be observed. Rheological spectra of other polymer concentrations are shown in Figure S2 of SI. Experiments were performed at 25 °C under nitrogen environment. Details about the experimental methods are reported in the SI.

The schematic representation of the polymer chains in the different concentration regimes is depicted in Figure 2. By assuming that the gel consists of entanglements and effective cross-links (Figure 2C), the mean–field percolation model developed by Langley^46−48^ can be invoked. In essence, the contributions of the entanglements and the cross-links to the total plateau modulus are additive:
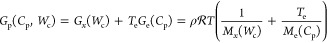
1where *G*_p_, *G*_e_, and *G*_*x*_ are the total, the entanglement (in dry conditions), and the cross-link plateau modulus, respectively.  is the universal gas constant, *T* is the absolute temperature, and *M*_e_ and *M*_*x*_ are the molar mass of entanglement strands and network strands, respectively. ρ is the density of the system. *T*_e_ is the entanglement trapping factor; the fraction of entanglements that are permanently trapped in the cross-linked network, with no possibility to undergo tube renewal. *T*_e_ changes from zero at the sol–gel transition (*W*_c_ = *W*_c,gel_) to unity at *W*_c_ ≅ 2*W*_c,gel_, with *W*_c,gel_ being the minimum water concentration needed to drive the gel formation of the systems. This leads to the fact that the entanglement contribution to the modulus of the network, *T*_e_*G*_e_, can be smaller than the entanglement modulus *G*_e_. That is, the comparison between the entanglement modulus of the entangled solution with no cross-links (dry solution) and that of a cross-linked network (wet solution) would corroborate whether *T*_e_*G*_e_ is smaller than or equal to *G*_e_, hence, whether *T*_e_ is smaller than or equal to 1. The experimental observations in Figure 1C (see high-frequency plateau of the *W*_c_ = 5 wt % gel) indicate that the contribution to the entanglement modulus in the network is always equal to or larger than *G*_e_, suggesting that the entanglements are already fully trapped. Thus, eq 1 can be rewritten as^40^
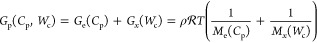
2Note that the details on how *T*_e_ grows beyond the sol–gel transition is not yet established.^40^ Once the polymer concentration is fixed, *G*_e_ can be obtained from the viscoelastic spectrum as the value of the storage modulus at the minimum of tan(δ) = *G*″/*G*′ in dry conditions (Figure S3 of the SI). The total plateau modulus of the gel can also be obtained experimentally the same way. Notably, the addition of water, at a constant *C*_p_, does not strongly affect *G*_e_, suggesting that the number of monomers in an entanglement strand (or *M*_e_) does not change considerably upon water addition or by essentially changing the solvent quality.^40^ Hence, under this simple idealization of the network, eq 2 could be used to extract the effective cross-link contribution (*G*_*x*_(*W*_c_)) to the total modulus, upon water addition.

**Figure 2:**
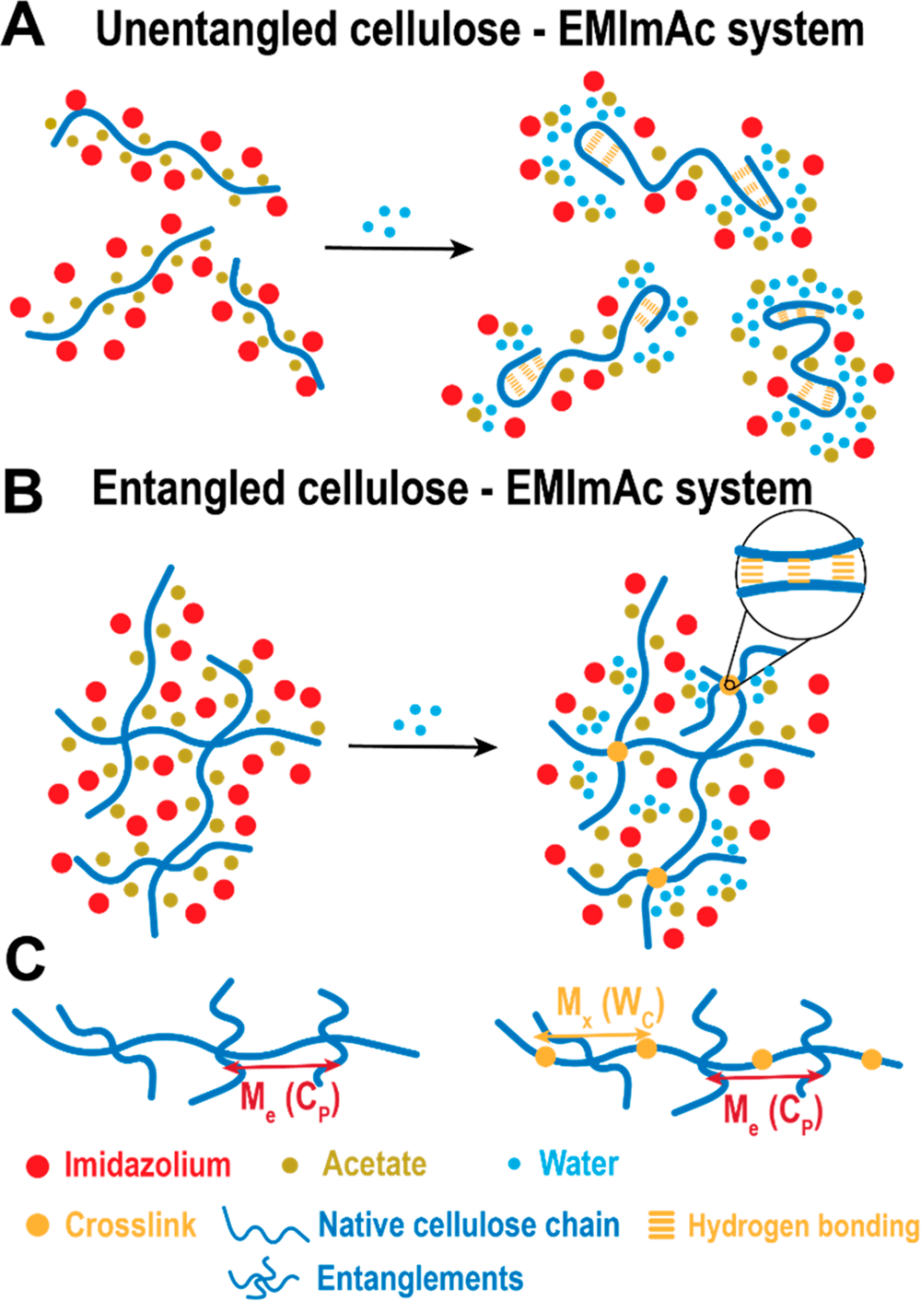
Schematic representation of intramolecular (A) and intermolecular (cross-links), (B) chain associations, and (C) a polymer network constituted by entanglements and cross-links, according to the rubber elasticity theory.^40^*M*_e_(*C*_p_) represents the molecular weight between two consecutive entanglements, and it varies with the polymer concentration *C*_p_, whereas *M*_*x*_(*W*_c_) is the molecular weight between two consecutive cross-links, and it varies with the water concentration *W*_c_.

The total plateau modulus *G*_p_ is depicted in Figure 3A against *W*_c_ at various polymer contents. Three regimes can be identified: (1) for water content less than 5 wt %, all the solutions behave like viscoelastic liquids, regardless of *C*_p_, with the modulus controlled by the entanglement molecular weight *M*_e_(*C*_p_). (2) As the water concentration increases between 5 and 15 wt % (depending on *C*_p_), a dynamic arrest can be observed, and a low-frequency plateau emerges. Intermolecular associations (cross-links) are now present in the system, though, the modulus is still controlled by the entanglement network and *M*_*x*_ > *M*_e_. (3) For larger water concentrations, *M*_*x*_ < *M*_e_ and the modulus was found to increase as *G*_p_ ∼ *W*_c_^3.0±0.2^, regardless of the initial polymer concentration. This goes beyond the linear dependence of the modulus on the cross-link concentration,^40^ suggesting a more complex network configuration compared to that schematized in Figure 2B,C. Indeed, various authors^49−53^ report that cellulose chains in solution, in the presence of water, form heterogeneous bundles or ordered mesophases, depending on the grade of cellulose used. Also, Tharmann et al.^54^ reported that the modulus of cross-linked actin gels varies with the power of 3.5 of the cross-linker concentration, and this was ascribed to the formation of actin chain bundles. Water may also affect the stiffness of the native cellulose chains, significantly influencing the modulus, as reported by MacKintosh and co-workers.^55^ However, it should be noted that the polymer content dependence of the modulus for dry solutions scales as flexible linear chains in good (or theta) solvent,^42^*G*_p_ ∼ *C*_p_^2.3^ (Figure S4 in the SI). The concentration dependence of the modulus of physically cross-linked gels has been largely discussed,^40,56−58^ and the accepted scaling for fully developed networks is *G*_p_ ∼ *C*_p_^2^ (others^58−60^ also proposed *G*_p_ ∼ *C*_p_^2.25^). That is, when the total plateau modulus is rescaled by *C*_p_^2^, for gels at *M*_e_ > *M*_*x*_, all the data collapse into a power–law with exponent 3.0 ± 0.2 (Figure 3B). This result leads to the scaling relation:

**Figure 3:**
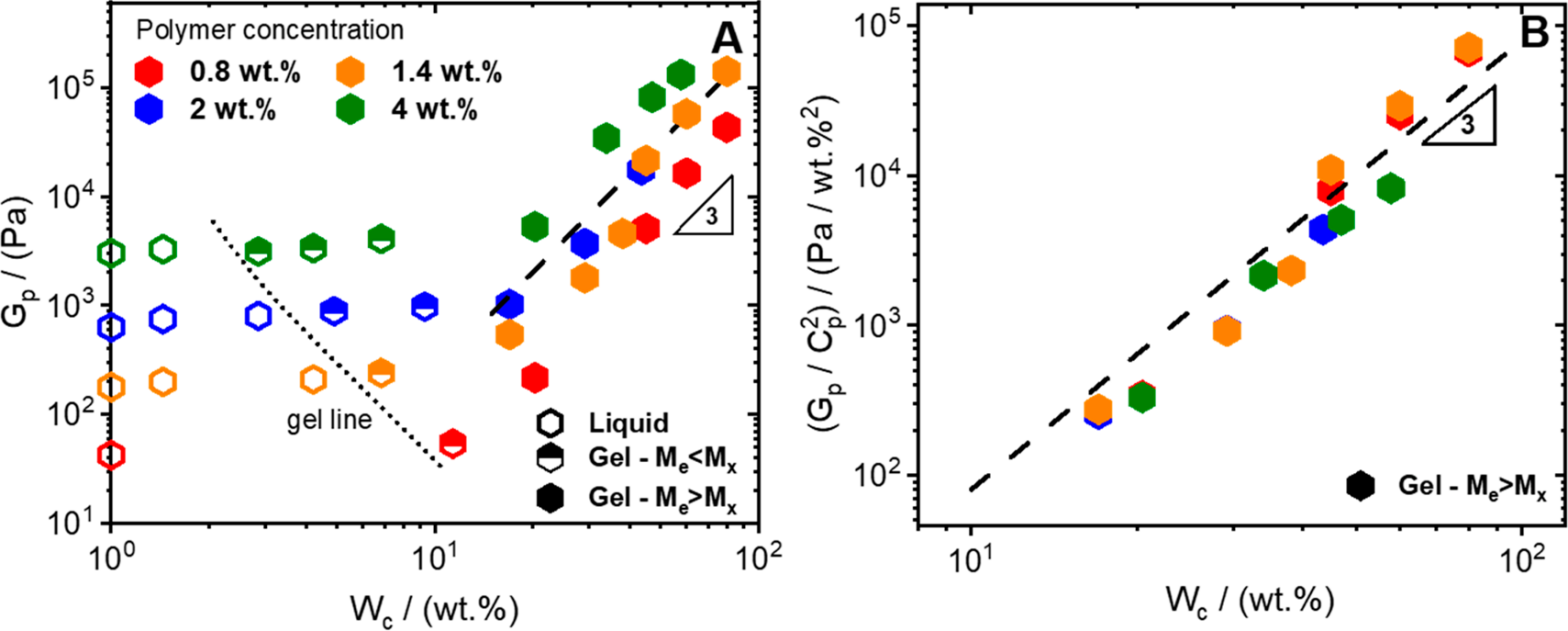
A) Plateau modulus G_p_ as a function of water concentration W_c_ for native cellulose/EMImAc solutions at various polymer concentrations (see legend). The dotted line indicates the region where the sol–gel transition is observed. Distinction is made whether the gel modulus is controlled by either the entanglements (half-filled symbols) or the cross-links (filled symbols). The data laying on the *y*-axis refer to dry condition, W_c_ = 0. B) Plateau modulus rescaled by the square of polymer concentration as a function of water concentration. The dashed lines in panels A and B represent the 3 ± 0.2 power-law dependence on water concentration, independent of the polymer content.


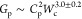
3

A progressive increase in *C*_p_ above *C*_e_ reduces the minimum amount of water needed to form a gel. In Figure 4A, the dynamic state diagram of native cellulose in EMImAc solutions is presented in terms of *C*_p_ vs *W*_c_. The water concentration at the gel formation, *W*_c,gel_, can be inferred, with a reasonably good approximation, from the linear viscoelastic spectra (Figures 1 and S2 of SI). Specifically, *W*_c,gel_ is the minimum water concentration needed to observe a dynamic arrest of the cellulose/IL solutions, with *G*′ > *G*″ over the entire frequency range 100–0.01 rad/s, for a given polymer concentration *C*_p_ > *C*_e_. *W*_c,gel_ is reported as a function of *C*_p_, normalized by *C*_e_, in Figure 4B. This representation clearly shows that when the polymeric system is barely entangled, the minimum *W*_c,gel_ is in the range 7–10 wt %, whereas as soon as more entanglements are formed (*C*_p_/*C*_e_ ∼ 5), a *W*_c,gel_ value as low as 3 wt % suffices to form a gel. In the measured polymer concentration region, the empirical power-law function *W*_c,gel_ = 11.4 × (*C*_p_/*C*_e_)^1±0.05^ can be used to estimate the critical water concentration for gel formation.

**Figure 4:**
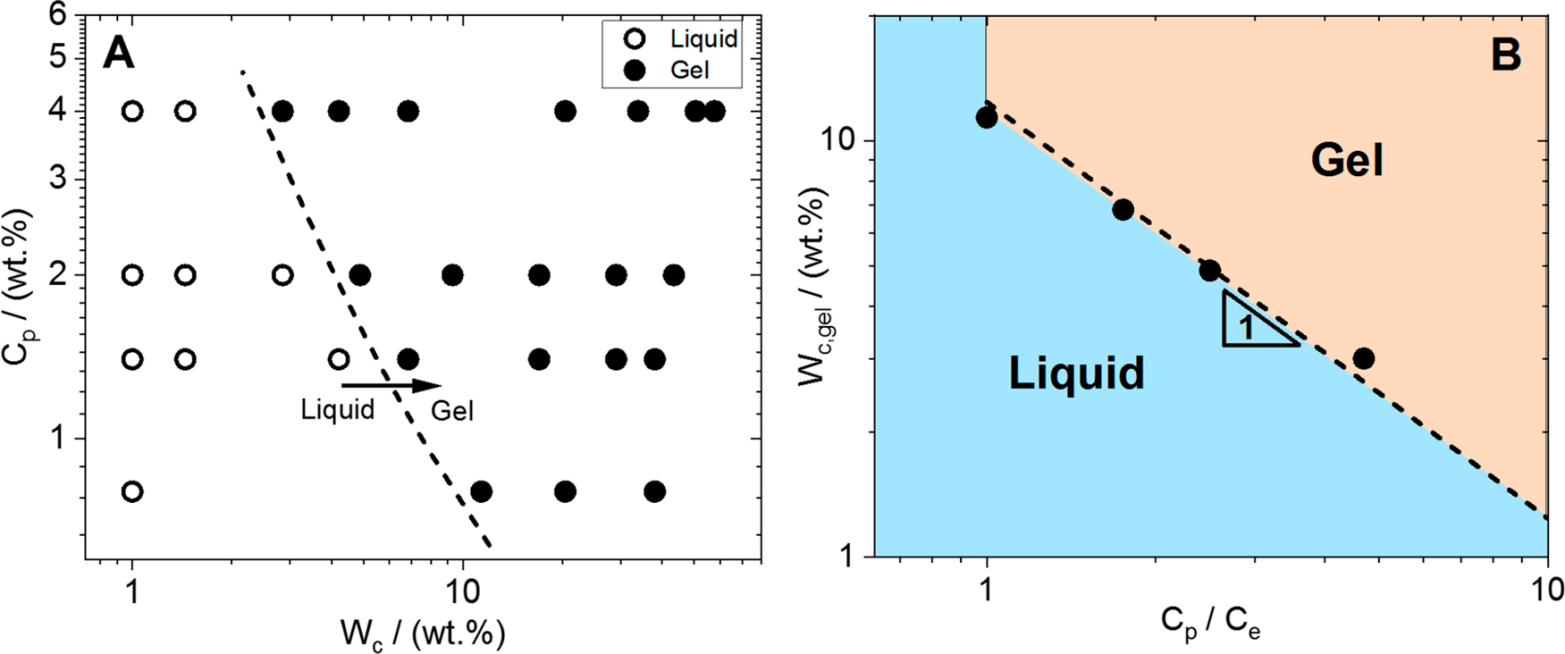
(A) Dynamic state diagram of native cellulose/EMImAc/water solutions in terms of polymer content (*C*_p_) against water concentration (*W*_c_). The dashed line and the horizontal arrow indicate the sol–gel transition. (B) Minimum water concentration for gel formation as a function of the polymer concentration (*C*_p_) normalized by the entanglement concentration (*C*_e_). The dashed line represents the best fit of the observed data to a power-law function, *W*_c,gel_ = 11.4 × (*C*_p_/*C*_e_)^1±0.05^. The origin of the equation is empirical and serves solely as an interpolating tool. The light blue- and orange-lighted areas mark the liquid and gel regions, respectively.

The following discussion delves into the mechanism and structure of the hydrogel formed via water–ionic liquid exchange. Figure 5A reports the Raman spectra for a 2 wt % solution of native cellulose in EMImAc, at various water concentrations. The Raman signal is exclusively produced by the ionic liquid: the latter and the dry solution spectra coincide (Figure 5A). While detailed atomistic vibrations of the EMImAc are reported elsewhere,^61^ the focus here is given to the O–C–O vibration of the anion (Figure 5B), occurring at a frequency of ∼900 cm^–1^. The progressive addition of water results in a horizontal shift of the O–C–O vibration peak toward higher Raman shifts or, equivalently, to smaller length scales. The O–C–O vibration peak position as a function of water concentration is reported in Figure 5C for various polymer contents. At first, the position of the O–C–O peak linearly increases with *W*_c_. In this regime, there is not yet enough water to bind to all acetate anions, so the peak reflects a combination of ionic liquid ion-pair and acetate-water interactions. As the water content increases, the Raman shift at the O–C–O peak attains a saturation value, reflecting the predominance of the anion–water interactions. The water–IL transition is reflected by the binding of the IL anions with water molecules, whose size is smaller than the ionic liquid cations, therefore resulting in a Raman shift that translates toward smaller length scales (or higher wave numbers, as can be seen in Figure 5C), as also described by Saha et al.^35^ Analogously, Shi and coauthors^62^ investigated water–EMImAc interactions, finding that, at low water content (1 wt %), the acetate anions are only coordinated by 0.25 water molecules, whereas at *W*_c_ = 5 wt % water, each acetate anion is coordinated by two water molecules. At higher water concentrations (*W*_c_ > 7 wt %), authors found that water–anion interactions predominate over the water–water and the water–cation ones. As the gel is dipped into water and remeasured, the Raman signal, solely due to the IL, is no longer detected (purple line in Figure 5A), suggesting a full or certainly substantial water–IL replacement. In addition to the horizontal shift, Raman spectra intensity decreases as the water is introduced to the system. This is due to the fact that the signal is solely produced by the IL, and when this is diluted or replaced by water, the Raman signal vanishes. In Figure 5D, the O–C–O stretching detected via FTIR (1383 cm^–1^) shows a similar shift toward higher wavenumbers as water is added to the system, pointing toward the strong interactions between the acetate anions and water (smaller) molecules. The same result was attained by Zhang and coauthors^63^ in starch/EMImAc/water solutions.

**Figure 5:**
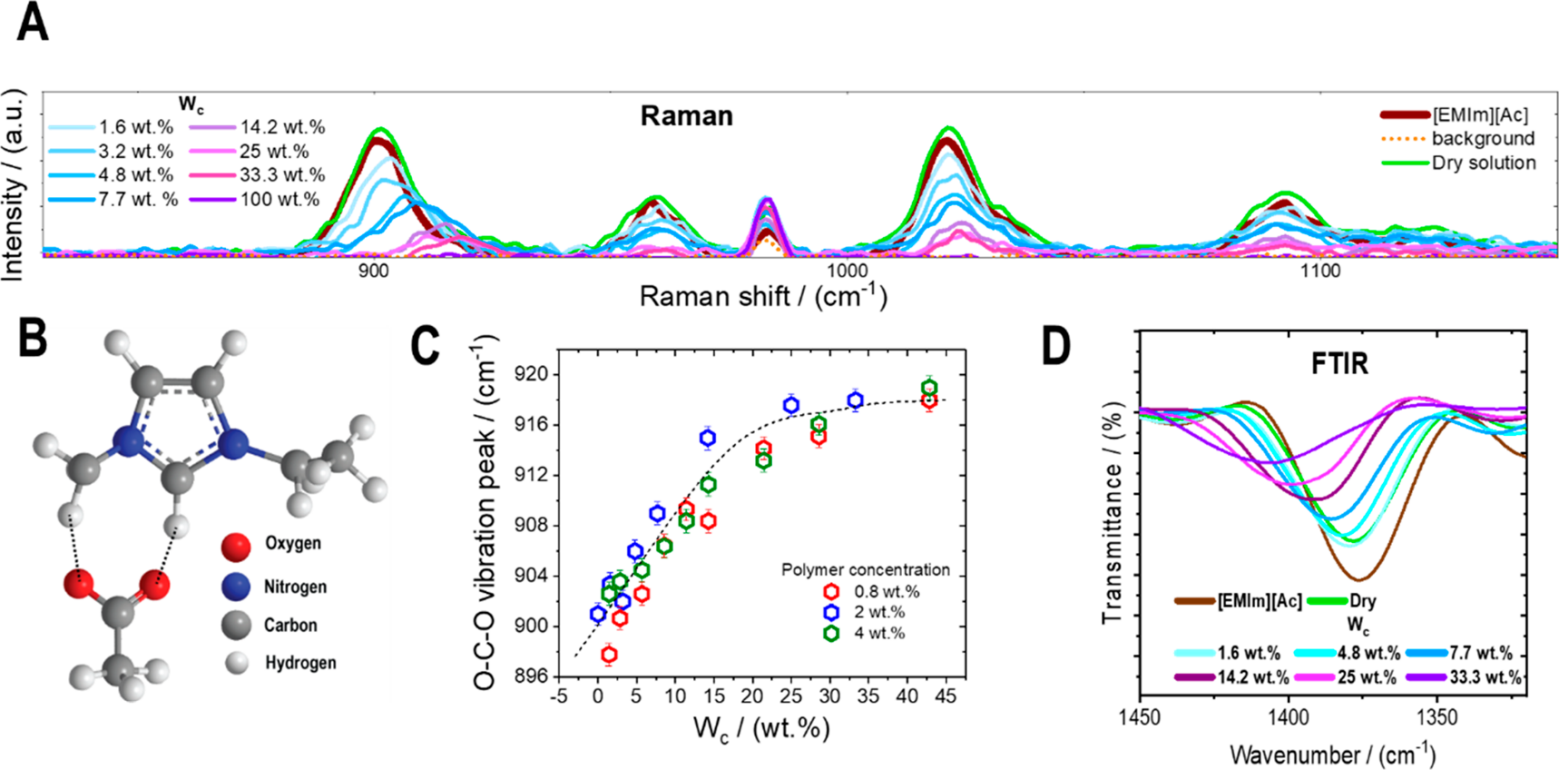
(A) Raman spectra for a 2 wt % native cellulose/EMImAc solution at various water contents (see legend); from dry to fully wet. Background and solvent signals (EMImAc) are also reported. Raman spectra were obtained at a wavelength equal to λ = 785 nm. Water does not provide any Raman signal in the Raman shift range explored. The O–C–O vibration of the anion, occurring at a frequency of ∼900 cm^–1^, shifts toward higher Raman shifts as the water content increases. (B) EMImAc chemical structure; [EMIm]^+^ at the top and [Ac]^−^ at the bottom. (C) O–C–O molecular vibration as a function of water concentration *W*_c_ for three different polymer concentrations (see legend). When the amount of water is not enough to bind to all of the IL anions, the peak position increases linearly with *W*_c_ and reflects both IL ion-pair and anion–water interactions. As the water content increases, the anion–water molecule interactions prevail, and the O–C–O peak position attains a saturation value that reflects the binding of the anion to a smaller molecule compared to the cation (e.g., water molecules). (D) FTIR for a 2 wt % native cellulose/EMImAc solution at various water content (see legend). The pure IL is also reported in the same panel. The experimental wavenumber window is narrowed to highlight the O–C–O stretch of the IL occurring at 1383 cm^–1^. All of the experiments were performed at 25 °C. Details about the experimental methods are reported in the SI.

The effect of water on native cellulose/EMImAc solutions is also distinct from the WAXS spectra. Figure 6A shows that the electron density of the ionic liquid dominates the scattering, since EMImAc and the dry solution exhibit similar scattering intensity curves. After the sample was submerged and kept in contact with a reservoir of water in a quartz X-ray capillary, the broad solvent diffraction peak shifts toward larger values, going from 16 to 19 nm^–1^, the latter being close to the first diffraction peak of bulk water observed at 25 °C, *q*_max,water_ = 20 nm^–1^.^64^ Consequently, the length scale associated with the WAXS peak, calculated as 2π/*q*_max_, varies from 0.39 to 0.33 nm, suggesting that the (smaller) water molecules partially replace the IL (bigger) ones, as conjectured via Raman and FTIR spectroscopy. As shown by Qiao and coauthors,^65^ the absence of sharp diffraction peaks in the WAXS spectra suggests not only the formation of a fully amorphous gel, but also a strong indication of good dissolution of cellulose in the ionic liquid.

**Figure 6:**
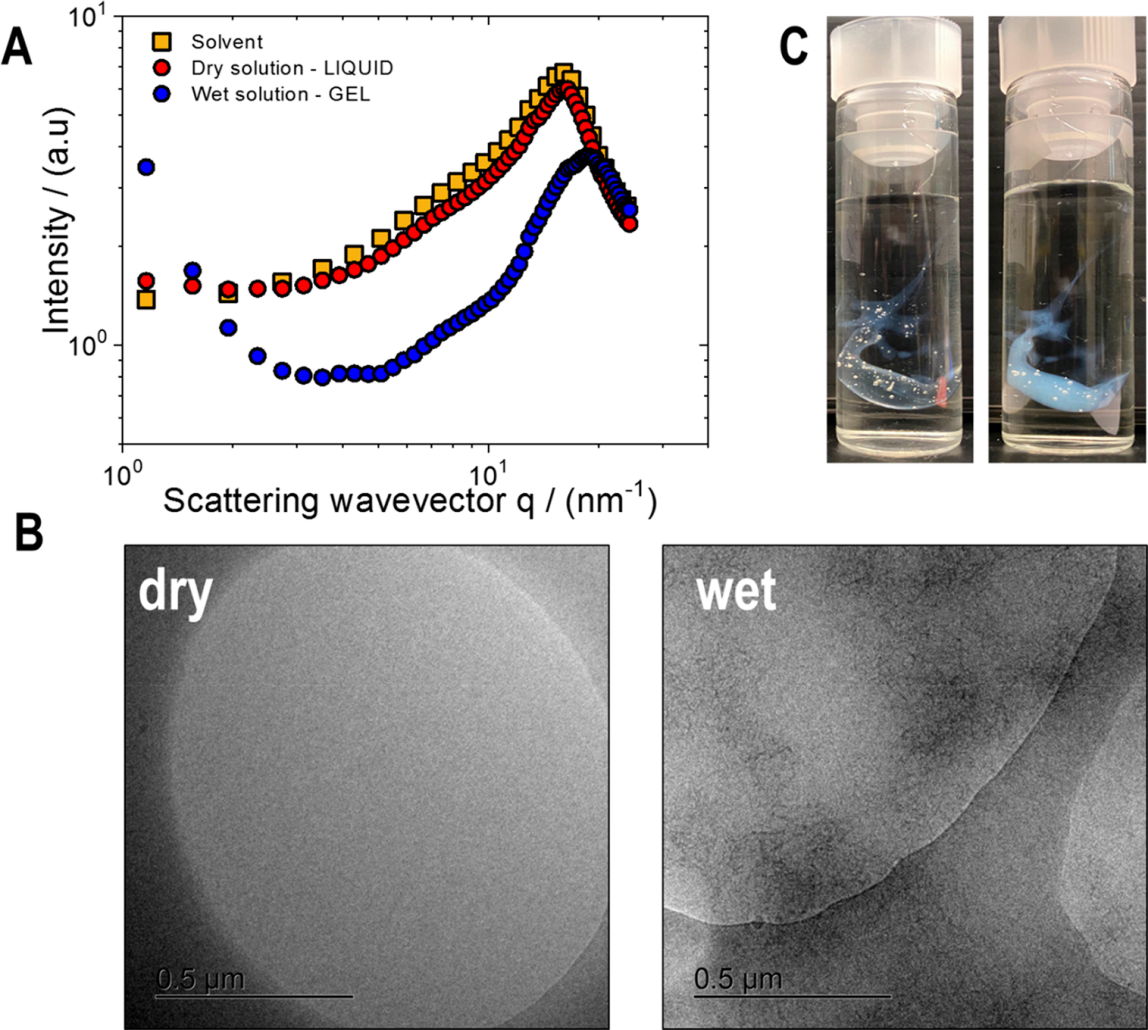
(A) Wide-angle X-ray (WAXS) scattering intensity, subtracted by the background scattering, as a function of the scattering wavevector for a 2 wt % native cellulose/EMImAc solution in dry state (red circles) and 20 days after the solvent exchange with water (blue circles). The intensity curve of the solvent (EMImAc) is reported as square symbols. (B) Cryo-TEM micrographs of a 2 wt % native cellulose/EMImAc solution in dry (left) and wet conditions (right). Scale bars: 0.5 μm. (C) Photographs of a submerged 2 wt % native cellulose/EMImAc solution in water, right away (left) and after 24 h (right) since water addition. Details about the experimental methods are reported in the SI.

Indeed, native cellulose dissolves at the molecular level in dry EMImAc, yielding a homogeneous solution, as observed via cryo-TEM in Figure 6B (left micrograph). However, when the solution is submerged in water and the gel is formed, the system becomes opaque/turbid (Figure 6C), pointing to a substantial solvent replacement that causes local heterogeneities of the refractive index. Note that the emerging high turbidity implies the formation of polymer aggregates.^66−68^ Indeed, our cryo-TEM images (Figure 6B, right micrograph) reflect the presence of aggregates of the order of half a micrometer and, together with the lack of crystallinity detected in WAXS, suggest that the formed hydrogels are strongly heterogeneous and amorphous. This can of course depend on the nature of the investigated cellulose system. Nevertheless, molecular dynamics simulations^6^ have shown that the addition of water to microcrystalline cellulose/ionic liquid solutions decreases the crystallinity.

New insights have emerged regarding the water-induced sol–gel transition in native cellulose–ionic liquid solutions. The formation of a gel is contingent on the presence of entanglements within the system, and its strength is elegantly regulated by the content of introduced water. At low water content, entanglements govern the gel elasticity, while at higher water concentrations, the modulus scales as *G*_p_ ∼ *C*_p_^2^*W*_c_^3.0±0.2^. This scaling goes beyond any “traditional” cross-linked network, and a possible conjecture lies its foundation on the reorganization of the cellulose chains into aggregates/bundles, as water is introduced to the solutions. This aspect will certainly trigger more studies toward the understanding of how natural polysaccharide chains interact and assemble in the presence of a nonsolvent. Both Raman and FTIR spectroscopy and WAXS support the hypothesis that water molecules, being smaller, replace the ionic liquid molecules, catalyzing the formation of intermolecular chain associations. In the hydrogel formed via such a water–ionic liquid exchange, the crystalline order is absent and, instead, polymer chain aggregates were observed. These findings hold significance in the design of native cellulose-based gels, offering a means to elegantly fine-tune their properties through the introduction of water.

## Supporting Information Available

Video 1: Dry native cellulose solution at 2 wt % of polymer content injected onto a Petri dish. The solution is a viscous liquid (MP4)
Video 2: Water addition onto the Petri dish and rapid water-IL exchange (MP4)
Video 3: Hydrogel formation: filament shown (MP4)
Materials; Shear rheology (specific viscosity measurements and additional frequency sweeps); Zimm model; Raman, cryo-TEM, and FTIR techniques (PDF)
