# Supplementary material for: Water-Driven Sol–Gel Transition in Native Cellulose/1-Ethyl-3-methylimidazolium Acetate Solutions
Source: ACS Macro Lett. 2024 Jan 29;13(2):219–26. doi: 10.1021/acsmacrolett.3c00710 (PMC10883029; doi:10.1021/acsmacrolett.3c00710)
Supplement: Supplementary file 4 — mz3c00710_si_004.pdf [file mz3c00710_si_004.pdf]

Supporting Information

Water-driven sol-gel transition in native cellulose/1-ethyl-3-methylimidazolium acetate (EMImAc) solutions

Roshan Akdar Mohamed Yunus[1], Marcus Koch[2], Philippe Dieudonné-George[3], Domenico Truzzolillo[3], Ralph H. Colby[4], and Daniele Parisi[1*]

[1]Engineering and Technology Institute Groningen (ENTEG), University of Groningen, Nijenborgh 4, 9747 AG Groningen, the Netherlands

[2]INM – Leibniz Institute for New Materials, Campus D2 2, 66123 Saarbrücken, Germany

[3]Laboratoire Charles Coulomb (L2C), UMR 5221 CNRS Université de Montpellier, Montpellier, France

[4]Department of Materials Science and Engineering, Penn State University, University Park, Pennsylvania 16802, United States

*E-mail: d.parisi@rug.nl

Table of Contents

Materials **2**

Shear rheology **2**

Zimm model **6**

Raman spectroscopy **7**

FTIR spectroscopy **7**

Wide-angle X-ray scattering **7**

Cryo-TEM **8**

Videos **8**

References **9**

Materials

The native cellulose was provided by Dow Incorporated (Midland, MI). Its molecular characterization and solution properties in various ionic liquids (ILs), including EMImAc, are reported elsewhere.[1,2] The ionic liquid solvent EMImAc, was purchased from abcr GmbH (Karlsruhe, Germany) with purity of ≥ 95% and CAS number 143314-17-4. The solvent was used directly without any further purification. The required amount of native cellulose was added to EMImAc and gently stirred with a spatula to ensure homogenous distribution and to prevent polymer powder getting trapped within the viscous solution during dissolution. Consequently, the sample was placed at 80°C for 60 minutes to yield clear cellulose/EMImAc solutions. Concentrations ranging between 0.1 wt.% to 4 wt.% were prepared. To prepare the ternary cellulose/EMImAc/water systems, to the above prepared cellulose/EMImAc solutions, required aliquots of water was added and gently stirred with a spatula.

Shear rheology

Rheological experiments were performed in a Discovery Hybrid Rheometer (HR-2) from TA Instruments (United States). 40 mm, 25 mm, and 8 mm diameter parallel plates were used depending on the viscosity of the samples. Nonlinear shear rheology experiments were carried out with a 25 mm diameter cone and plate geometry with cone angle equal to 1°. For the dry samples, the solutions were annealed at 80 °C for 20 minutes prior to the measurements. The solutions were loaded into the rheometer, and dynamic strain sweeps were executed at 100 rad/s to determine a strain within the linear viscoelastic (LVE) regime. Consequently, frequency sweeps were performed over a range of frequencies varying from 100 to 0.01 rad/s. All the measurements were carried out at 25 ºC and in the presence of nitrogen.

Figure S1 reports the specific viscosity (η_{sp}) as a function of the polymer concentration (C_p) in dry state at 25 °C. The crossover concentrations obtained from the specific viscosity scaling[3] served to identify both the overlap (C*) and the entanglement (C_e) concentrations.

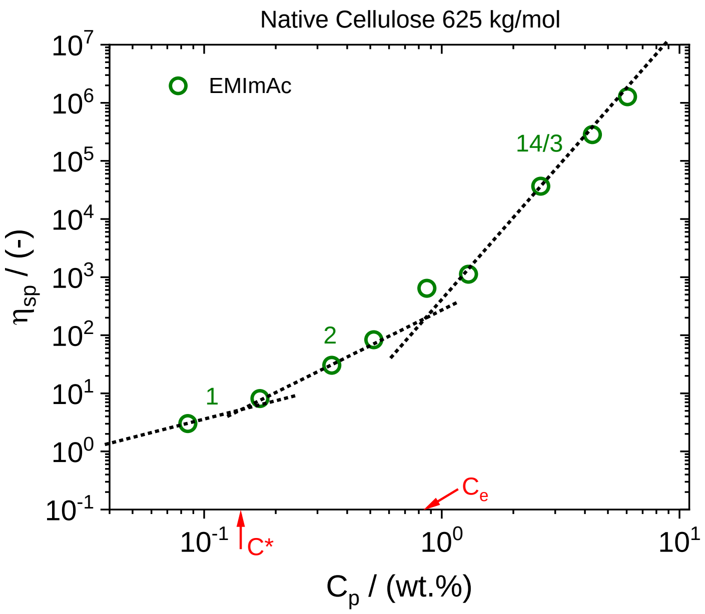

Figure S1. Specific viscosity as a function of mass concentration for native cellulose solutions in EMImAc at various concentrations and at 25 °C. Black dotted lines are a guide for the eyes. The red arrows indicate approximately where the characteristic change of slope occurs between the dilute-to-semidilute regime (C*) and the semidilute-to-entangled regime (C_e).

Figure S2 depicts the rheological spectra in terms of storage G' and loss G" modulus as a function of the oscillation frequency ω, for native cellulose/EMImAc solutions at various polymer and water content. We remind here that that a water-driven gel can be formed only when the polymer concentration C_p is equal or larger than the entanglement concentration C_e.

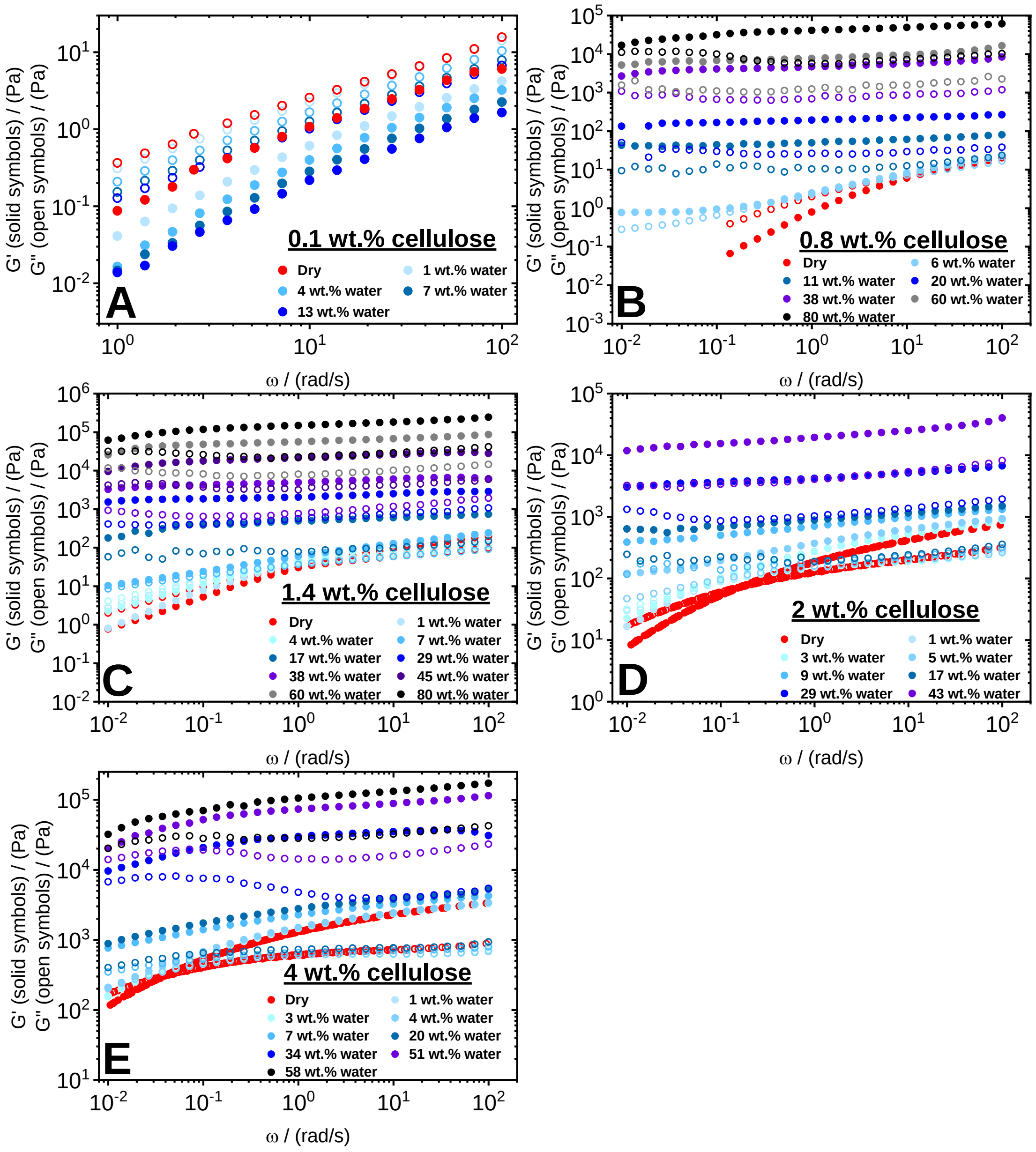

Figure S2. Storage G′ (closed symbols) and loss G″ (open symbols) modulus as a function of oscillation frequency ω for native cellulose/EMImAc solutions at various polymer and water mass fractions. The polymer concentration C_p is C^* (0.1 wt.%, panel A), C_e (0.8 wt.%, panel B), 1.4 wt.% (panel C), 2 wt.% (panel D) and 4 wt.% (panel E), with the last three concentrations being within the entanglement regime. The water content is reported in the legend of each panel. Experiments were performed at 25 °C.

Figure S3 reports the viscoelastic spectra in terms of loss factor tan(δ) = G''/G' as a function of oscillation frequency ω, for native cellulose/EMImAc solutions at various polymer and water content. This representation was used to estimate the plateau modulus, either from the minimum of the loss factor or from the storage modulus value at the highest frequency, when a clear minimum was not attained.

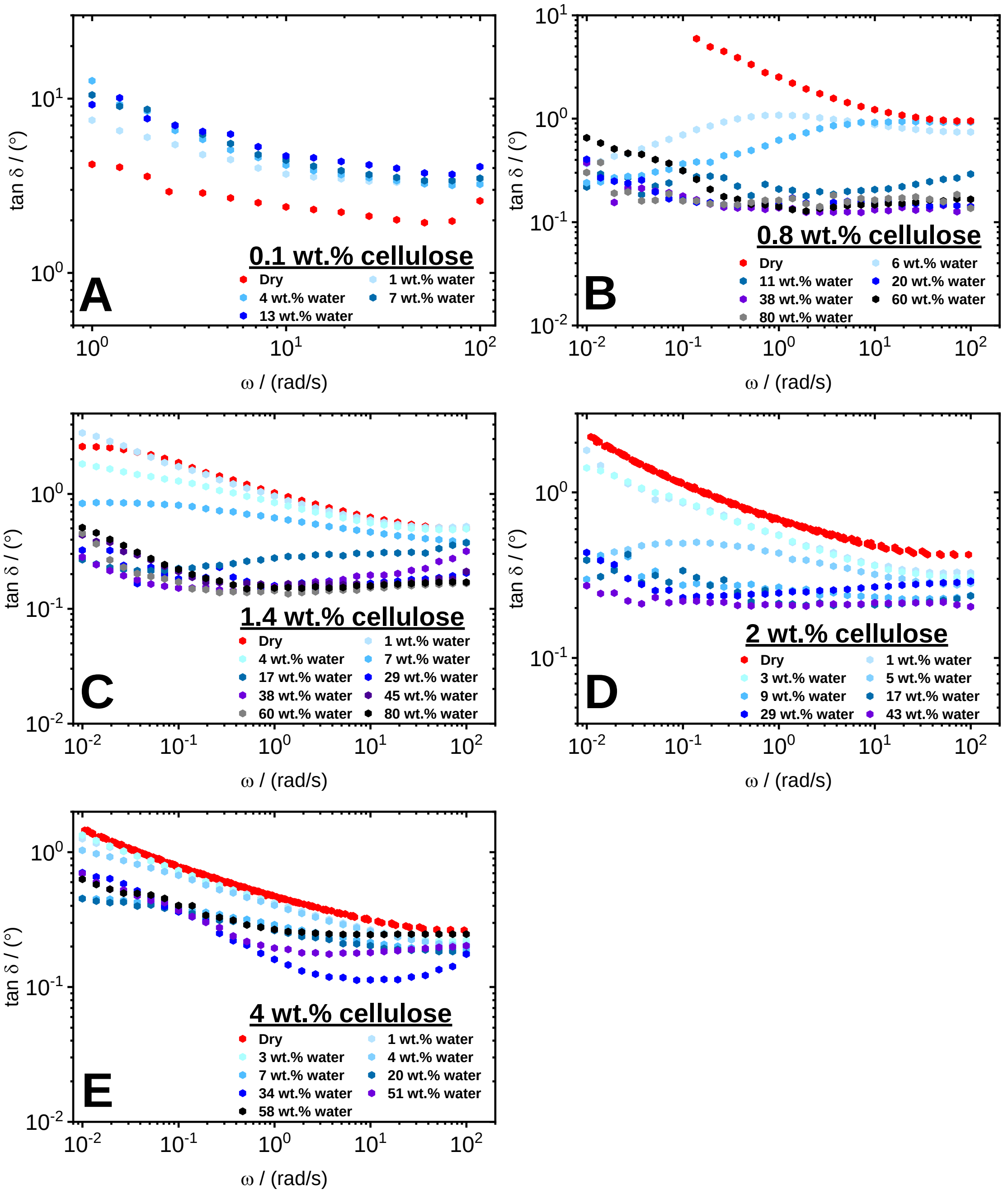

Figure S3. Loss tangent tan(δ) = G''/G' as a function of angular oscillation frequency ω for native cellulose/EMImAc solutions at various polymer and water mass fractions. The polymer concentration C_p is C^* (0.1 wt.%, panel A), C_e (0.8 wt.%, panel B), 1.4 wt.% (panel C), 2 wt.% (panel D) and 4 wt.% (panel E), with the last three concentrations being within the entanglement regime. The water content is reported in the legend of each panel. Experiments were performed at 25 °C.

Figure S4 shows the polymer concentration dependence of the plateau modulus for dry native cellulose/EMImAc solutions. The observed 2.3 power-law agrees well with the theoretical predictions for entangled flexible linear polymer solutions, in good or theta conditions.[3]

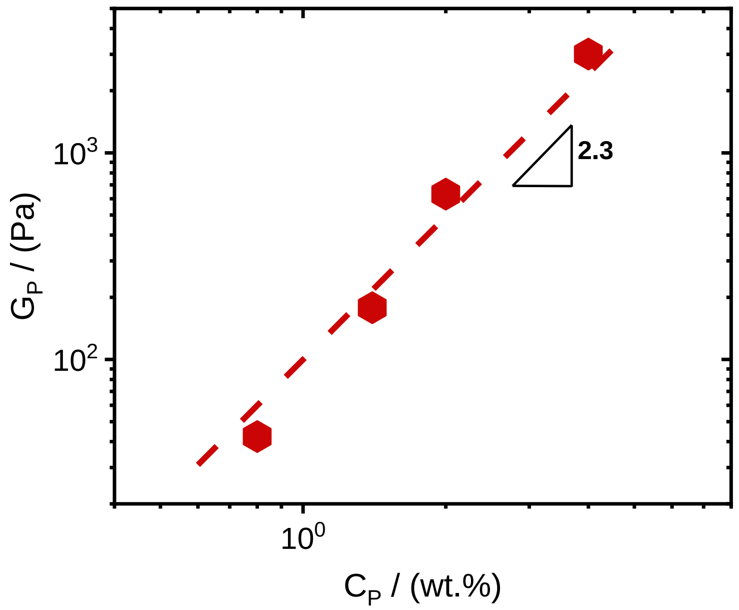

Figure S4. Plateau modulus G_P as a function of polymer concentration C_P for dry native cellulose/EMImAc solutions. The dashed line represents the 2.3 power-law dependence on polymer concentration that is expected for entangled flexible polymer solutions.[3]

Zimm model

The Zimm model is a renowned molecular model to describe the dynamics of flexible linear polymer solutions in the nonentangled regime.[3] According to the Zimm model, the polymer chain diffuses of a distance of the order of its own size during the time τ_Z:

$$\tau_Z \sim \frac{\eta_s}{kT} \quad \text{(S1)}$$

where η_s is the solvent viscosity, kT the thermal energy and R the polymer chain size. On the other hand, the contribution of the polymer content to the viscosity in the Zimm (or Rouse) model can be expressed as:

$$\eta - \eta_s \sim kT \frac{\phi}{Nb^3} \tau_Z \quad \text{(S2)}$$

With η being the viscosity of the solution, ϕ the polymer volume fraction, N the degree of polymerization of the chain, and b the Kuhn monomer length. By substituting Eq. S1 into Eq. S2, and dividing both sides by η_s and ϕ, it is possible to obtain the following scaling law:

$$\frac{\left(\frac{\eta}{\eta_s}-1\right)}{\phi} = \frac{\eta_{sp}}{\phi} \sim \frac{R^3}{Nb^3} \quad \text{(S3)}$$

where η_{sp} is the specific viscosity. In the present case, the viscosity of the solutions reported in Figure 1A was calculated as G''/ω in the terminal region in both dry and wet conditions. The viscosity of the solvent was estimated via the logarithmic mixing rule:

$$ln(\eta_s) = w_{EMImAc}\ln(\eta_{EMImAc}) + w_{water}\ln(\eta_{water}) \quad \text{(S4)}$$

Where w is the mass fraction either of water or ionic liquid, respectively, as indicated in Eq. S4. The viscosity of pure water and EMImAc at room temperature was considered equal to 1 mPa.s, and 93 mPa.s, respectively. Note that, as reported in the literature,[4] the mixtures of EMImAc and water are far from being ideal, and their interactions are much more complex. The linear mixing rule can practically work only up to ~ 10 wt.% water, and the use beyond such value would lead to wrong estimation of the total viscosity. Finally, as the ratio $\frac{\eta_{sp}}{\phi}$ is proportional to the cube of the size of the polymer chain, a reduction of the same with increasing water concentration translates into a reduction of the polymer chain size (see inset in Figure 1A of the main text).

Raman spectroscopy

Raman spectra were obtained in a Rheo-Raman ThermoFisher setup (Haake Mars 60 rheometer + iXR Raman Spectrometer) with a laser at λ = 785 nm wavelength. The aperture was set to 50 μm slit, grating 400 lines/mm, estimated resolution 2.3-4.3 cm^{-1} and Raman shift range between 3357-6 cm^{-1}. All the experiments were performed at 25 °C on native cellulose/EMImAc solution at C_p = 2 wt. %, at various water concentrations.

FTIR spectroscopy

Fourier-transform infrared (FTIR) spectra were obtained using a Shimadzu IR Tracer-100 spectrophotometer, capturing transmittance spectra between the 500–4000 cm^{-1} range. The data were collected at a resolution of 4 cm^{-1}, averaging 64 scans for each sample. Experiments were performed at room temperature on a native cellulose solution at 2 wt.% of polymer content and increasing water concentration as reported in the main text.

Wide-angle X-ray scattering

WAXS measurements were conducted in an in-house setup located in Laboratoire Charles Coulomb, University of Montpellier. The system featured a high-brightness, low-power X-ray tube paired with an aspheric multilayer optic, specifically the GeniX^{3D} from Xenocs, which provided an ultralow divergent X-ray beam (0.5 mrad, λ = 0.15418 nm). To achieve a clean 0.6

mm beam diameter and a flux of 35 million photons per second at the sample, scatterless slits were employed. The experiment was performed in a transmission configuration, and scattered intensity was captured by a 2D "Pilatus" detector by Dectris (490×600 pixels) with a pixel size (area) of 172×172 μm^2. The detector was positioned at 1.9/0.2 meters from the sample. The sample was loaded into cylindrical quartz capillary tubes with a 1 mm diameter and sealed with wax. The scattering intensity was collected across a scattering wavevector range of 1 nm^{-1} to 20 nm^{-1}. All the experiments were performed at constant room temperature T = 22.0 ± 0.5°C. All recorded intensities were corrected for transmission, and the contribution from the empty capillary was subtracted.

Cryo-TEM

Two samples were prepared for cryo-TEM. For the dry sample at C_p = 2 wt.%, a 2 µl droplet of the solution was placed on a holey carbon supported copper grid (Plano, Wetzlar, Germany, type S147-4), blotted for 2 s and vitrified in undercooled liquid ethane using a Gatan (Pleasanton, OR, United States) CP3 plunge-freezer. For the wet sample C_p = 2 wt.%, a 2 µl droplet of the solution was placed on the holey carbon film and 5 µl of water was added over it for 30 min before plunge-freezing. The samples were transferred to a Gatan model 914 cryo-TEM sample holder under liquid nitrogen and visualized using a JEOL (Akishima, Tokio, Japan) JEM-2100 LaB6 TEM at 200 kV accelerating voltage under low-dose conditions (CCD camera Gatan Orius SC1000, 2 s acquisition time).

Videos

#1. Dry native cellulose solution at 2 wt. % of polymer content injected onto a Petri dish. The solution is a viscous liquid.

#2. Water addition onto the Petri dish and rapid water-IL exchange.

#3. Hydrogel formation: filament shown.

References

(1) Utomo, N. W. Rheology of Native Cellulose in Ionic Liquids. MSc thesis, PennState University, State College, 2019.

(2) Utomo, N. W.; Nazari, B.; Parisi, D.; Colby, R. H. Determination of Intrinsic Viscosity of Native Cellulose Solutions in Ionic Liquids. *Journal of Rheology* **2020**, *64* (5), 1063–1073.

(3) Rubinstein, M.; Colby, R. H. *Polymer Physics*; Oxford university press New York, 2003.

(4) Hall, C. A.; Le, K. A.; Rudaz, C.; Radhi, A.; Lovell, C. S.; Damion, R. A.; Budtova, T.; Ries, M. E. Macroscopic and Microscopic Study of 1-Ethyl-3-Methyl-Imidazolium Acetate–Water Mixtures. *J. Phys. Chem. B* **2012**, *116* (42), 12810–12818.
